# Ecology and Management of a Large Outbreak of Avian Botulism in Wild Waterbirds in Northeastern Italy (2019–2022)

**DOI:** 10.3390/ani14162291

**Published:** 2024-08-06

**Authors:** Stefano Volponi, Maria Alessandra De Marco, Roberta Benigno, Enea Savorelli, Matteo Frasnelli, Laura Fiorentini, Giovanni Tosi, Lia Bardasi, Elena Toschi, Roberta Taddei, Roberto Cocchi

**Affiliations:** 1 Wildlife Service, Institute for Environmental Protection and Research (ISPRA), 40064 Ozzano dell’Emilia, BO, Italy; stefano.volponi@isprambiente.it (S.V.); roberto.cocchi@alice.it (R.C.); 2 Office of Sustainability Education and Animal Welfare, Environmental and Land Protection Service, Comune di Ravenna, 48124 Ravenna, Italy; robertabenigno@comune.ravenna.it; 3 Animal Health–Ravenna, Department of Public Health, Azienda Unità Sanitaria Locale della Romagna, 48124 Ravenna, Italy; enea.savorelli@auslromagna.it; 4 Istituto Zooprofilattico Sperimentale della Lombardia e dell’Emilia Romagna “B. Ubertini” (IZSLER), 25124 Brescia, Italy; matteo.frasnelli@izsler.it (M.F.); laura.fiorentini@izsler.it (L.F.); giovanni.tosi@izsler.it (G.T.); lia.bardasi@izsler.it (L.B.); elena.toschi@izsler.it (E.T.); roberta.taddei@izsler.it (R.T.)

**Keywords:** avian botulism, outbreak, ecology, management, Southern Europe, Italy, ecosystem change

## Abstract

**Simple Summary:**

In all continents, except in Antarctica, avian botulism outbreaks occur in wild waterbirds with different recurrences and a high severity. This feared disease is due to the ingestion of botulinum neurotoxins (BoNT) mainly produced by *Clostridium botulinum*, also able to produce persistent spores representing an efficient form of environmental resistance while waiting for suitable conditions enabling bacterial vegetative growth. In addition, concomitant ecological and environmental changes (e.g., the presence of decaying organic material of plant and animal origins) represent a suitable substrate for the replication of *Clostridium* spp. strains producing BoNT in conditions of high temperatures and the absence of oxygen. This study describes the occurrence, evolution, and management of a severe outbreak of botulism that occurred in a protected wetland area of northeastern Italy, where over 2000 waterbirds (mostly carcasses, and only to a lesser extent sick birds) were recovered in 2019. We also describe the activities underlying the avian botulism monitoring and management performed in this area in 2020, 2021, and 2022. According to an ecological approach, anthropogenic changes that may trigger the occurrence of avian botulism in wetland habitats should be carefully assessed, and habitat management actions should be adaptively planned to protect the biodiversity of these vulnerable ecosystems, which are increasingly affected by ongoing global change.

**Abstract:**

Avian botulism is a paralytic disease due to the ingestion of botulinum neurotoxins (BoNT) produced by anaerobic, sporigenic bacteria (notably, *Clostridium botulinum*). Wild waterbirds worldwide are affected with variable recurrence and severity, and organic material decaying in wetland habitats may constitute a suitable substrate for the replication of clostridia strains producing BoNT in conditions of high temperatures and the absence of oxygen. Here, we describe a large outbreak of avian botulism that occurred in the Valle Mandriole protected area of northeastern Italy (VM). After the recovery in late summer of a few duck carcasses that molecularly tested positive for BoNT-producing clostridia, in October 2019, the avian botulism escalation led to a total of 2367 birds being recovered (2158 carcasses and 209 sick birds). Among these, 2365/2367 were waterbirds, with ducks accounting for 91.8% of the total (2173/2367) and green-winged teals representing 93.5% of the ducks. After the quick collection of dead and sick birds (from 4 to 11 October 2019) and the flooding of the VM wetland (from 5 to 12 October 2019), the 2019 botulism emergency apparently ended. Following two water inputs in May and July 2020, only one pooled sample obtained from 16 bird carcasses found that year in VM tested positive for clostridia type C by real-time PCR, whereas, after to the implementation of measures deterring the bird’s presence, new avian botulism cases—due to clostridia type C and C/D, according to molecular and animal-model tests of confirmation—led to the collection of 176 waterbirds (82 carcasses and 94 sick ducks) and 16 waterbirds (9 carcasses and 7 sick ducks) in the summers 2021 and 2022, respectively. In conclusion, the prevention, management, and control of the disease rely on habitat management, the quick and careful collection/removal of animal carcasses, and the regular monitoring and surveillance of live and dead birds.

## 1. Introduction

Botulism is a feared neurological disease reported in humans and animals and is due to botulinum neurotoxins (BoNT) produced by anaerobic, sporigenic bacteria belonging to the genus *Clostridium*, mainly represented by *Clostridium botulinum.* Of the nine types of BoNT identified so far, the A, B, E, and F are responsible for human botulism [1], whereas the C and the mosaic variant C/D are most frequently identified in wild birds [2]. The pathogenesis of avian botulism is not completely clear in wildlife [2], but, as assumed in poultry [3], both the ingestion of preformed toxins [4,5] and the in vivo production of toxins by clostridia might coexist during botulism outbreaks in wild birds [2]. Notably, cases of type C botulism have been confirmed in 264 bird species belonging to 39 families, including 22 waterbird families [1,2,6].

Avian botulism is associated with a severe neuro-paralytic syndrome, which represents an important cause of death affecting wild aquatic birds in all continents, except in Antarctica [2,6]. As reviewed in detail by Wobeser [7], this pathological condition has long been known in North America for the recurrence and extreme severity characteristics of outbreaks involving as many as tens of thousands of waterbirds, with an estimated mortality of 250,000 and 200,000 ducks at the Great Salt Lake in 1932 and Pakowki Lake in 1995, respectively. According to the available literature, the first major outbreaks of avian type C botulism reported in Central Europe occurred in Poland, causing an estimated number of 5500 and 1600 birds being affected in 2011 and 2012, respectively [8]. As recently reviewed by Gutiérrez-Arnal et al. [2], twenty avian botulism outbreaks were reported worldwide from 2014 to 2023 in the USA, Canada, Chile, Argentina, Spain, the UK, Australia, and New Zealand.

This paralytic and often fatal disease is caused by the action of potent BoNT, mainly produced only by toxigenic strains of *C. botulinum*, considered by many as a saprophytic bacterium [9] that is widespread in the soil of freshwater and marine habitats, and also present in wetlands where avian botulism is not reported [4,10,11]. The spores produced by this anaerobic bacterium can persist for years in a quiescent state of life [2,9], representing for *C. botulinum* an efficient form of environmental resistance while waiting for suitable conditions enabling the vegetative growth of the BoNT-producing bacterium. Moreover, an increasing risk of botulism outbreaks in wetlands is closely linked to the concurrent high temperatures and the absence of oxygen in decaying materials [1,6]. In this context, the direct ingestion of *C. botulinum* replication substrates or of invertebrates fed on these substrates—such as carrion fly larvae capable of concentrating neurotoxins produced in decomposing carcasses [12,13]—represent possible sources of intoxication of numerous waterbird species, showing varying degrees of vulnerability [14].

In Italy, fifteen outbreaks of type C botulism were reported between 1973 and 2011 in birds and mammals from 7 of the 20 Italian regions, with the highest number of outbreaks (equal to 5) and dead birds (equal to 96) occurring in the Emilia-Romagna Region of Northern Italy [15]. Moreover, two type C/D botulism outbreaks involving 136 feral waterbirds and at least 20 wild waterbirds occurred in 2009 in an urban park of Central Italy and in 2017 in a protected wetland area of Southern Italy, respectively [1,16].

Our study describes the occurrence, evolution, and management of the severe outbreak of avian botulism that emerged in 2019 in a protected area of northeastern Italy. We also describe the activities underlying the avian botulism monitoring and management performed in this area in 2020, 2021, and 2022.

## 2. Materials and Methods

### 2.1. Study Area and Routine Activities of Environment Management

**Figure 1:**
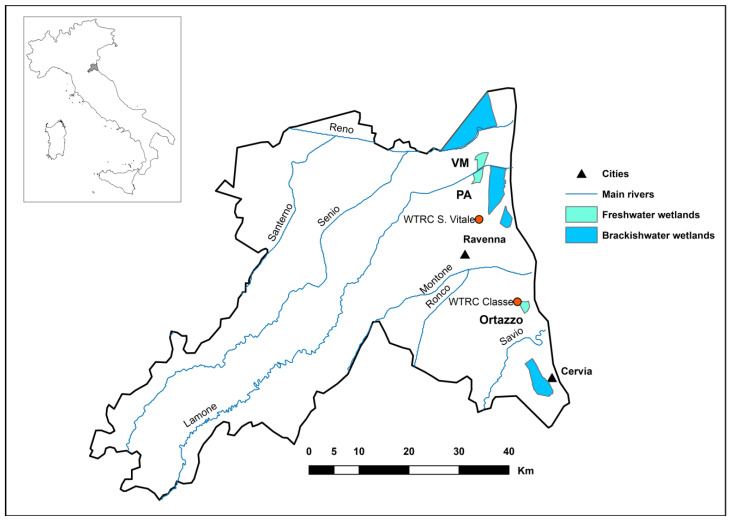
Study area map including the Valle Mandriole (VM) and Punte Alberete (PA) protected areas (Ravenna Province, Emilia Romagna Region, northeastern Italy).

The Valle Mandriole, herein referred to as VM, otherwise known as “Valle della Canna” (i.e., wetland of the reed), is a freshwater coastal wetland of 271 hectares located (44°32′09″ N, 12°13′28″ E) near the mouth of the Lamone River, 10 km north of the city of Ravenna (Emilia Romagna Region, northeastern Italy). Together with the nearby twin area of Punte Alberete (187 hectares, 44°30′56″ N, 12°13′13″ E), herein referred to as PA, VM is the last remnant of the vast ancient marshes, which, between the beginning of the XIX century and the mid-XX century, extended over a progressively smaller surface area of 8000 hectares to the north-west of Ravenna. The long-term historical reclamation ended in the early 1970s with the construction of the final stretch of the Lamone river’s outlet to the sea and the complete embankment of VM. These two protected areas are part of a complex of coastal wetlands (Figure 1) located between the cities of Ravenna and Comacchio and are composed of some contiguous enclosed brackish lagoons (ca. 13,000 hectares) and—dispersed in the surrounding countryside—a plethora of small basins (0.5–5 ha) used as water reserves for agriculture practices and for waterfowl hunting [17,18].

VM is fully protected as part of the Regional Park ”Delta del Po Emilia-Romagna” [19] (herein referred to as Park) and is included in the Ramsar List of Wetlands of International Importance and in the Natura 2000 network as IT4070001-ZSC-ZPS [20,21,22,23]. The property is shared by several public bodies, and although this wetland is largely owned by the Emilia-Romagna Region (243 hectares out of 271), the Comune di Ravenna (herein referred to as the Municipality of Ravenna) has always maintained the direct management of VM, particularly when, up to 1996, it was used as a water storage basin of the civil aqueduct [18].

VM has an average depth of approximately 1.0 m, with an overall capacity of about 2,430,000 cubic meters, which was raised up to a depth of 1.5–2.0 m, for an overall capacity of 5,000,000 cubic meters, when the basin was used as a water reservoir [18,21]. Until the beginning of the 1990s, the aquatic plants were abundant and the ecosystem was healthy and appeared as a typical freshwater swamp at the mid-stage between the coastal lagoons closest to the sea and the flooded forests of the areas furthest from the mouth. The water was clear, with small areas of open waters covered by white water lily (*Nymphaea alba*) and extensive submerged meadows of rigid hornwort (*Ceratophyllum demersum*), spiked water-milfoil (*Myriophyllum spicatum*), and bladderwort (*Utricularia australis*), surrounded by extensive reed beds of common reed (*Phragmites australis*) mixed with narrow-leaved cattail (*Typha angustifolia*) and common club-rush (*Schoenoplectus lacustris*), interspersed by patches of hygrophilous grey willow (*Salix cinerea*) shrubs and white willow (*Salix alba*) [24,25].

The avifauna living in VM was rich and diverse, typical of freshwater wetlands. About 30 species included in the Annex II of the Bird Directive (Directive 2009/147/EC) and more than 50 other migratory species were reported [26,27,28]. Among ducks, the mallard (*Anas platyrhynchos*), common pochard (*Aythya farina*), gadwall (*Mareca strepera*), and ferruginous duck (*Aythya nyroca*) were regular breeders, with the last one having a large majority of the Italian breeding population here [26,29]. The thick reedbeds hosted a diverse community of wintering and breeding passerines including species with fragmented distribution such as the bearded reedling (*Panurus biarmicus*), Savi’s warbler (*Locustella luscinioides*), and mustached warbler (*Acrocephalus melanopogon*), as well as little bitterns (*Ixobricus minutus*) and a heronry with grey herons (*Ardea cinerea*), purple herons (*Ardea purpurea*), and white herons (*Ardea alba*). The floating leaves of the water lily supported an important colony of whiskered terns (*Chlidonias hybrida*), amounting to 50% of the Italian population [30,31].

Being, together with the close PA area, the only protected freshwater marsh closed for hunting, during autumn migration and winter, VM is still a resting spot and a refuge for many species of ducks, geese, waders, and flamingos (*Phoenicopterus roseus*) [32]. Bird numbers vary according to water levels and climatic conditions as well as in relation to the presence of birds in the entire wetland area of the coastal strip of the Po delta. The green-winged teal (*Anas crecca*), mallard, northern shoveler (*Spatula clypeata*), common pochard, and greylag goose (*Anser anser*) are the more common and abundant species in mid-winter [32,33].

In 1996, the use of VM as a water storage basin for the aqueduct was discontinued, and the regular maintenance of the hydraulic system, water levels, and aquatic vegetation was also terminated. As a result, over the next few years, there was a rapid deterioration of the total environment. This happened while, in the meantime, the wetland has been given greater environmental protection, as proposed by the Natura 2000 SCI/ZPS, and the Park Authority was directly entrusted with the management and conservation of its biodiversity [18]. The severity of the habitat changes and fast environmental degradation of VM emerged dramatically at the beginning of the new century. In 2001, five years after abandonment, the aquatic plants practically disappeared, and in 2004, three years after the mass death of the aquatic vegetation, the water appeared rotten and populated by extensive bacterial colonies [18]. The death of the reed bed plants and the collapse of the willow patches completely changed the landscape, transforming VM in a huge uniform basin. The community of reedbed passerines was greatly impacted and only reduced numbers of the two most common species (*Acrocephalus scripaeus* and *Acrocephalus arundinaceus*) continued to nest, while the seven colonial species of herons and egrets, great and pygmy cormorants (*Phalacrocorax carbo* and *Microcarbo pygmaeus*, respectively), Eurasian spoonbills (*Platalea leucorodia*), and glossy ibises (*Plegadis falcinellus*) nesting in the huge mixed heronry were forced to move, mostly in the close PA marsh {31]. These habitat modifications were well documented [17] and visible from the sequence of historical aerial photographs [18,31]. After 2004, VM was declared by ARPAE Emilia-Romagna (i.e., the regional environmental agency) “unsuitable for fish life”, pursuant to Legislative Decree 152/2006, due to the very high concentration of ammonia due to the putrefaction of the tons of organic substances (aquatic plants, invertebrates, fish, bird excrements) lying on the bottom of the partially anoxic wetland.

**Figure 2:**
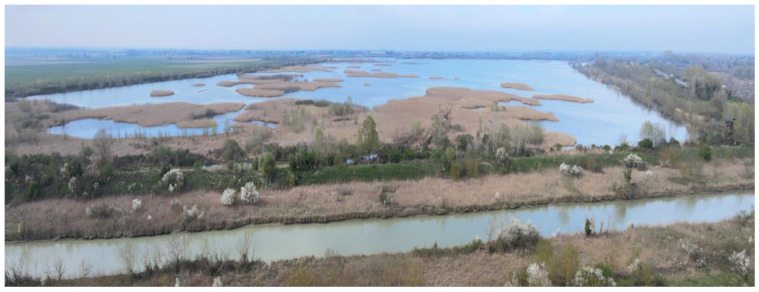
Aerial image of Valle Mandriole taken in late winter, when the water levels are at their highest. A segment of the Lamone River flows south of the wetland. A birdwatching tower is visible on the right edge of the photo (Ravenna Province, Emilia Romagna Region, northeastern Italy).

Since 2011, the Municipality of Ravenna has been carrying out routine maintenance on the VM. This involves completely draining the basin during the summer months of July to September and refilling it in October with water from a canal connected to the Reno River, a few kilometers to the north [21]. The VM water levels are at the highest during the winter period (Figure 2).

### 2.2. Samples and Laboratory Analyses

During the suspected avian botulism emergency, the following samples were collected and submitted to the Forlì and Ravenna laboratories of the Istituto Zooprofilattico Sperimentale della Lombardia e dell’Emilia-Romagna “B. Ubertini” (herein referred to as IZSLER) for analyses:

- 2019—A total of 30 wild bird carcasses, found in VM, including 11 unspecified ducks, 17 pied avocets (*Recurvirostra avosetta*), 1 feral pigeon (*Columba livia domestica*), and 1 eurasian magpie (*Pica pica*), from 5 September to 9 October;
- 2020—A total of 1 great cormorant and 1 mallard found dead in VM and PA wetlands, on 17 June and 13 July, respectively, and 1 flock of 16 unspecified duck carcasses found in VM on 19 August;
- 2021—A total of 28 waterbird carcasses, including 24 unspecified ducks, 2 green-winged teals, 1 mallard, 1 unspecified ardeid found in VM, and 10 environmental samples from VM (water and water with sediment) between 18 August and 25 August;
- 2022—A total of 16 waterbirds carcasses (including 8 mallards, 3 mute swans, *Cygnus olor*, 4 northern shoveler, and 1 green-winged teal) found in the PA and VM wetlands between 4 May and 12 August.

According to standard procedures, the eligible samples were tested by molecular methods for the detection of pathogens potentially causing neurological signs and mortality in wild birds, i.e., Influenza A Virus (IAV) [34] and Newcastle Disease Virus (NDV) [35]. The IAV-positive samples were further tested by PCR methods specific for H5 and H7 avian influenza virus (AIV) subtypes [36,37,38].

The samples were further processed according to standard procedures to be tested at the Bologna laboratories of the IZSLER (Regional Reference Laboratory for the Diagnosis of Botulism) by multiplex real-time PCR (RT-PCR) for the presence of *Clostridium* spp. genes capable of producing botulinum toxins type A, B, E, F, C, and D and their mosaic variants C/D and D/C according to standard procedures [39,40,41,42]. Briefly, liver and intestine samples were 10-fold-diluted in pre-reduced Fortified Cooked Meat Medium (FCMM) broth and incubated in anaerobic conditions at 30 °C for a maximum of 96 h. Then, 1 ml of each enrichment broth was subjected to DNA extraction by Chelex 100 Chelating Resin (Bio-Rad, Hercules, CA, USA), and four multiplex Realtime PCR schemes were carried out for genes encoding toxin types A and B, E and F, C and D, and CD and DC, respectively. Before DNA extraction, each sample was supplemented with 100 cells of a C. botulinum type B–like strain—a strain that had naturally lost the gene encoding the toxin—as an internal process control (PC). The isolation of BoNT-producing clostridia from molecularly positive samples was not performed in this study.

A limited number of samples was also processed for botulinum toxins detection via a mouse bioassay [43] (see Section 3.2 for details).

## 3. Results

### 3.1. Emergence, Evolution, and Management of Avian Botulism

#### 3.1.1. Summer and Autumn 2019: Chronicle of Events

1. Suspect and Confirmation of Avian Botulism—On 5 September, a few ducks were found dead in VM, and some of them were collected by the staff of the local Wildlife Treatment and Rehabilitation Center (herein referred to as WTRC) to be submitted to the Forlì laboratories of the Istituto Zooprofilattico Sperimentale della Lombardia e dell’Emilia-Romagna “B. Ubertini” (herein referred to as IZSLER) to investigate the cause of death. Promptly, the laboratory tests revealed the presence of BoNT-producing clostridia (see Section 3.2 for details).

Three weeks later (on 27 September 2019), hunters reported symptomatic ducks showing signs of flaccid paralysis in the hunting ponds adjacent to VM. Although the hunting season for ducks and other migratory birds had started on 15 September, apart from these cases, no other reports were received by the Municipality. To promptly verify whether the problem originated in the neighboring ponds or in VM, local police officers from the Municipality’s Environmental and Land Protection Service were sent to the affected areas to investigate the presence of dead animals and report the outcome of the patrol rounds.

2. Planning of Measures to Counteract the Outbreak Escalation—On 2 October, several dead or symptomatic birds were reported, and carcass collection was planned to send further samples to the IZSLER (Territorial Section of Forlì) for analyses. The day after, due to the escalation of the health emergency, the Municipality of Ravenna established a Technical Table of experts, healthcare and local administration personnel, and stakeholders who were to analyze the situation to acquire key elements for promptly implementing suitable, urgent interventions by coordinating all the bodies and subjects involved in the avian botulism emergency (Technical Appendix A).

The outcomes from the Technical Table first meeting—attended by delegates representing the Ravenna Municipality, Park, WTRC, veterinary service of the Azienda Unità Sanitaria Locale (AUSL) della Romagna, Emilia-Romagna Agriculture, Hunting and Fishing Service, Carabinieri Command for Biodiversity and Parks Protection, Ravenna Servizi Industriali society (RSI), Romagna Acque society, and Azimut society—included the following evidence-based recommendations: (1) organize the collection of dead and symptomatic birds; (2) organize the disposal of carcasses and the hospitalization of living individuals at the local WTRC; and (3) plan an emergency water input from the Reno river by RSI to increase the water level and oxygenate the wetland, even if the alternative of possibly drying the whole wetland was not fully ruled out at this time. Thus, RSI agreed to provide a week of water input (from 5 October to 12 October) with an injection of 65,000 m^3^/day, equivalent to approx. 2 cm per day, enabling the potential shift from the current level of 5 cm under the mean sea level to 10 cm a.m.s.l. The RSI pointed out that this exceptional measure implied a very large effort and a heavy economic impact for the society itself.

**Figure 3:**
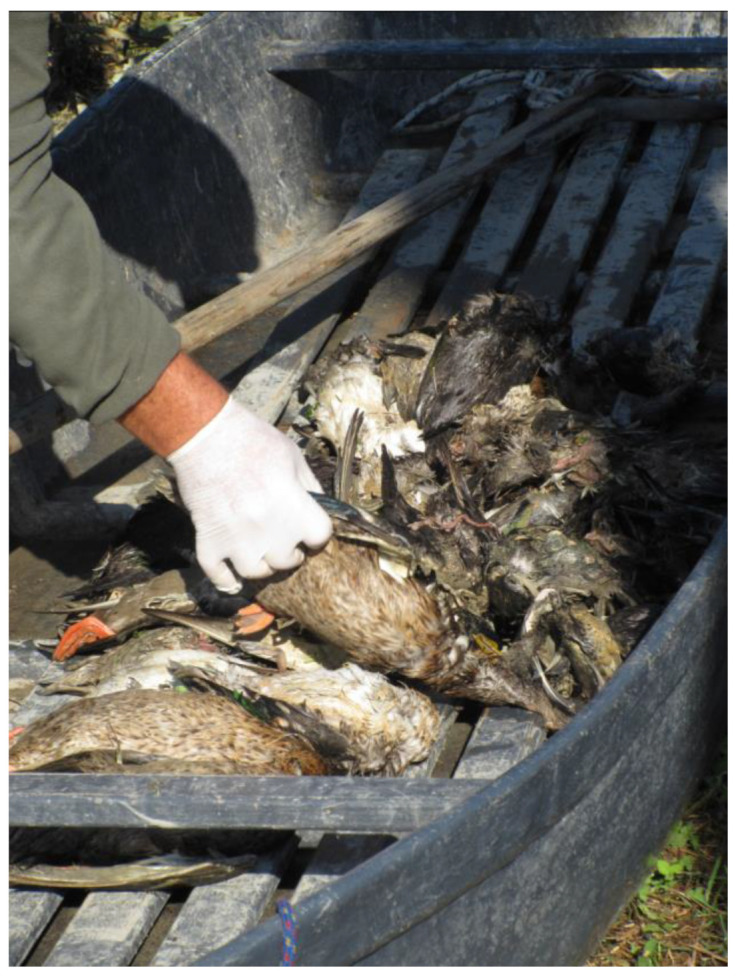
Waterbird carcass collection during the large outbreak of avian botulism occurred in October 2019 in Valle Mandriole. The collection, also including sick birds, was carried out using a small flat-bottom boat suitable for moving in the very shallow water of the wetland. Green-winged teals and mallards are shown in the image (Ravenna Province, northeastern Italy).

3. Implementation of the Planned Management Measures—The activities aiming to search, find, and remove many intoxicated animals started in VM on 4 October, when well over a thousand carcasses and some hundreds of symptomatic birds were collected (see Section 3.3 for details). In detail, a twenty-member team was assembled and informed/trained by the Local Municipal Policy to jointly carry out the planned activities with local hunting organizations and volunteers. To improve efficiency in field activities, the team was divided into ten two-unit patrols, each of which had a so-called “battana”, that is, a small flat-bottom boat suitable for moving in the very shallow water of the wetland (Figure 3).

In fact, this sort of punt boat allowed the operators to lean out from a seated position and pick up (also using a hand net) bird carcasses more easily and ill birds suffering from flaccid paralysis more difficultly.

**Figure 4:**
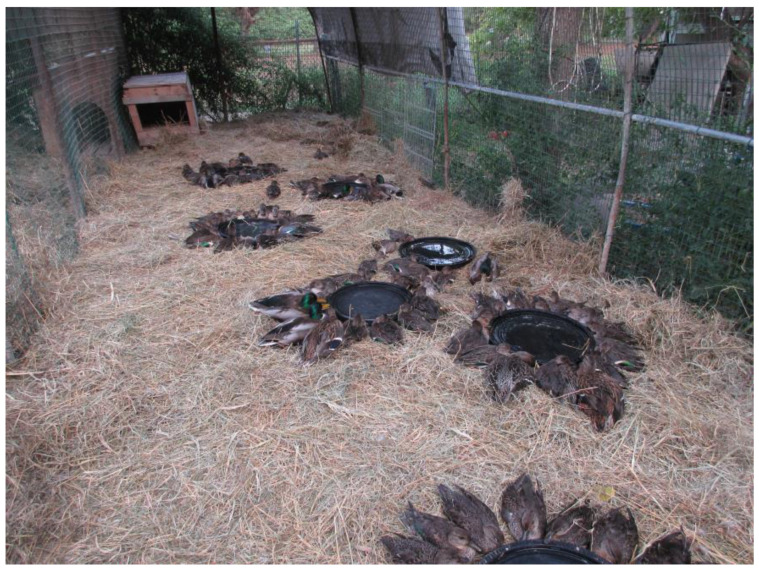
During the large outbreak of avian botulism that occurred in October 2019 in the Valle Mandriole wetland, sick waterbirds were taken to the local Wildlife Treatment and Rehabilitation Center, where rehydration and antibiotic therapy were administered. Green-winged teals, mallards, and northern shovelers are shown in the image (Ravenna Province, northeastern Italy).

According to the veterinary service of the AUSL of Romagna, precautional measures including the use of rubber gloves, as well as the ban on eating, drinking, and smoking, were implemented while handling the affected animals. During the severe die-off, the rescued birds were given initial care consisting of carrying the hypothermic and soaked ducks to a set collection point, where they were rehydrated with NaCl 0.9% solution and treated with antibiotic therapy consisting of the oral administration of gentamicin sulfate solution at a dose of 25 mg/kg once daily. Then, the ducks were placed in sunshine on a van box to dry up before being taken to the WTRC (Figure 4), where rehydration and antibiotic therapy were continued for a time ranging from 8 to 10 days. The overall rate of survival was 65%.

At the same time, the bird carcasses collected were counted, examined where possible for species identification, and placed in suitable plastic bags. A few carcasses were sent to the IZSLER for diagnostic investigations (see Section 3.2 for details), while the rest of the birds that died in VM, and those that died at the WTRC, were disposed by incineration in a plant authorized under current legislation (EC Regulation No 1069/2009).

Still, on 4 October, due to a significant waterbird mortality attributable to BoNT intoxication, ISPRA (i.e., the Italian Institute for Environmental Protection and Research) expressed a favorable opinion to an NGO’s request, supporting the temporary closure of any form of hunting activity within a radius of at least 3 km from the perimeter of the VM area. On 7 October, the President of the Po Delta Park deliberated the immediate temporary suspension of the hunting activity, as suggested by ISPRA. Some days later, the decision was refined to resize the banned area to the full boundaries of the wetlands in the range of 3 km and to allow for the hunting of terrestrial species.

**Figure 5:**
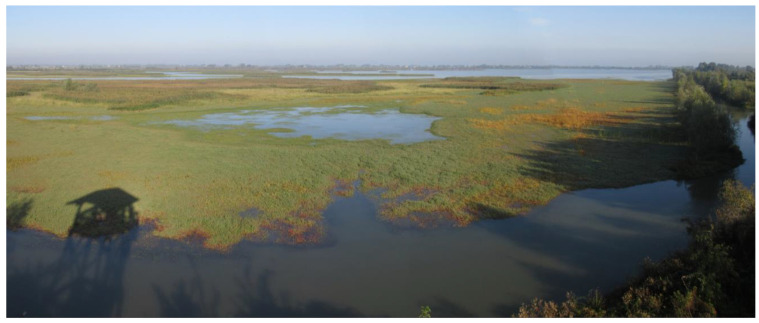
Status of the Valle Mandriole protected area during the large avian botulism outbreak that occurred in October 2019. Image taken on 8 October 2019 from the birdwatching tower located in the south-eastern corner of the wetland (Ravenna Province, northeastern Italy).

Meanwhile, the searching, finding, and removing of the intoxicated birds continued in VM, accounting for the collection of some hundreds of carcasses and a few dozen symptomatic birds on 8 October, followed on 11 October by the collection of a few dozen and less than ten dead and ill birds, respectively (see Section 3.3 for details). The VM area during the botulism outbreak is shown in Figure 5.

4. Toward the End of the 2019 Emergency—On 11 October, the second meeting of an enlarged Technical Table, established by the Municipality of Ravenna, was held to discuss the emergence and evolution of the avian botulism outbreak. On the same day, the personnel of ISPRA, Park, and Ravenna Municipality carried out a joint inspection in VM that aimed to census alive birds and search and collect dead and ill birds.

On 13 October, due to the scheduled and no longer deferable maintenance needs of the distribution network, RSI stopped the flow of water coming from the Reno River into the VM.

On 17 October, the Park Authority requested ISPRA’s opinion about the revocation of the closure of waterbird hunting in the wetlands surrounding the VM area. On 25 October, ISPRA expressed a conditional favorable opinion regarding the above revocation, provided that the revocation took place when the maximum temperature stabilized in the Ravenna coastal area below 20 °C for at least seven days, according to conditions considered sufficient to inhibit the larval development of insects on carcasses or other biological material present on site [44]. On 4 November, respecting the ISPRA’s technical–scientific opinion cited above, the Park Executive Committee revoked the suspension of hunting activity, which was resumed on 7 November.

#### 3.1.2. Spring and Summer 2020: Chronicle of Events

Already in the early spring, due to scarce winter rainfalls, the depth of the water above mean sea level was considerably low in VM (20 cm a.m.s.l.) and significantly reduced in comparison to the minimum level agreed upon by the management protocol (30–35 cm a.m.s.l.).

The hydraulic works aimed at providing a sufficient flow of water from the Reno River were suspended early between February and March 2020 due to restrictions related to the COVID-19 emergency, which hindered the procurement of materials needed to complete the water pipes and sluice gates.

As a result, work completion could not be guaranteed before the summer 2020, when temperatures and evapotranspiration would be the highest and water availability would be minimal for VM due to low river flows and increased irrigation demand.

To prevent the risk of avian botulism, in early April, the Park authority suggested management measures including the full summer drying of VM. Alternatively, the ISPRA, the Municipality of Ravenna, the Emilia-Romagna Region Park Service, and the head of the local NGO formerly charged with the PA and VM habitat management considered drying too risky and recommended not carrying out any summer drying but instead keeping VM constantly flooded (further details available upon request). Eventually, it was agreed to maintain the highest water levels through the frequent input of fresh water and to perform constant surveillance for the early detection of any suspect case of avian botulism. These measures were consistent with either the decision of the local judicial authority—who initiated a legal proceeding for the environmental disaster and seized a large part of VM affected by botulism in the autumn of 2019—and the anti-COVID-19 restrictions, which did not allow for involving large technical staff and volunteers in field operations needed to remove large quantities of dying fish and/or search and retrieve ill birds, as accomplished during the previous botulism event.

At the end of May and in July 2020, the RSI granted the input of several hundred cubic meters of water, allowing for keeping water levels high enough during the whole summer. Some suspected cases of avian botulism were reported in VM and PA between June and August 2020, and the waterbird carcasses were sent to the IZSLER (Territorial Section of Forlì) for laboratory analyses.

#### 3.1.3. Summer 2021: Chronicle of Events

On 1 July, the Ravenna Municipal Environmental Police started conducting daily inspections at VM to promptly detect any potential cases of avian botulism. Efforts were made to maintain water levels between 30 and 10 cm a.m.s.l. with the input of 1,374,800 cubic meters of water from the Reno River from June to early August. However, the water temperature had risen considerably, leading to the visible development of algal bloom, as typical for eutrophic freshwater wetlands facing oxygen-poor conditions that may enhance the risk of avian botulism [45].

On 17 August, the water level was low in VM (15 cm a.m.s.l.), and the first signs of an incoming botulism event were reported in the same area that was affected by the severe crisis of 2019. Two recently deceased ducks were collected in the morning and sent for analysis to the IZSLER (see Section 3.2 for details). On the same day, more than 50 ducks displaying symptoms of BoNT intoxication were promptly treated by the municipality’s veterinary staff with saline solution and antibiotics and taken to the local WTRC. Similar to that described for the 2019 emergence of avian botulism (Section 3.1.1) a large-scale operation was then arranged to search for and promptly remove and dispose of carcasses, as well as collect sick birds. In the afternoon, a task force consisting of Municipality of Ravenna and Park personnel, along with volunteers, retrieved dozens of carcasses and intoxicated birds (see Section 3.3 for details). In addition, the VM managers decided to open the water sluice to promote the rapid and complete desiccation of the wetland. This measure enabled the fish to move spontaneously towards the emissary and facilitated the removal of a significant portion of the fish biomass, which could serve as an ideal food source for the multiplication of *Clostridium* spp.

On 18 August, about forty dead or sick waterbirds were found and collected. To keep waterbirds away from the contaminated area, five gas cannons were strategically placed and operated at various points in VM. On 19 August, two more gas cannons were added, while the outflow of water and the search and collection of sick and dead birds continued.

On 20 August, the water level of the main basin was close to 0 cm a.m.s.l., and water was only present in the canals that have an average depth of 25 cm a.m.s.l. On 20, 21, and 22 August, a few live birds were recovered (see Section 3.3 for details). On 22 August, approximately 500 kg of fish, mainly carps (*Cyprinus carpio*), were recovered alive from drying pools and channels and released into the Fossatone Canal.

On 23 August, the inlet of new freshwater from the river Reno and the simultaneous discharge of decaying waters started to allow for water exchange in the VM channel. On 25 August, the last search for sick birds was carried out: seven alive and two dead waterbirds were collected. No dead or sick birds were found in the following days.

On 10 September, VM re-flooding operations began by introducing water from the Reno River. As BoNT were detected on August 20 in the residual water (see Section 3.2 for details), the drainage gates were left open during the initial outflow to allow the remaining river water to flow out through the main channel, thus keeping the fish alive. After verifying the replacement, the discharge gates were closed, and the wetland began to be filled. It will take a few weeks for the water to reach the optimal levels for that period.

#### 3.1.4. Spring and Summer 2022: Chronicle of Events

On 4 May, seven juvenile ducks were found dead at the PA wetland—in areas previously involved in the recent avian botulism cases—and submitted to the IZSLER for diagnostic tests (final part of this section). In the summertime, surveillance activities linked to the recent drought and warm weather conditions were implemented, leading to the recovery, on 3 August, of one dead juvenile swan, which was sent to the IZSLER for diagnostic analyses. However, the avian botulism emergency was triggered on 9 August, when four ducks showing symptoms of avian botulism were observed at VM and promptly recovered by the environmental police, to be taken to the WTRC and treated as previously described (Section 3.1.1 and Section 3.1.3). Due to the low water level (15 cm a.m.s.l.), to prevent the spread of avian botulism, it was deemed essential to desiccate the wetland by opening the drain on the Rivalone Canal. On 10 August, similar to that described for the 2019 and 2021 emergencies (Section 3.1.1 and Section 3.1.3), teams of volunteers and personnel from the Ravenna Municipality entered VM to search for sick birds and bird carcasses. On 10 and 11 August, one duck and two ducks were respectively recovered and taken to the WTRC. The ongoing desiccation of the VM wetland led to a rapid decrease in the water level from the initial 15 cm a.m.s.l. to 12 cm, 10 cm, and 3 cm a.m.s.l. on 10, 11, and 12 August, respectively. From 3 August to 12 August, nine waterbird carcasses were collected and submitted to the IZSLER for diagnostic tests (final part of this section). When the search for symptomatic birds continued in the following days, along with the desiccation of the wetland, no other sick or dead birds were found.

### 3.2. Avian Botulism Confirmation by Laboratory Tests

- When tested by Real-Time PCR [34,35] for the presence of Newcastle disease and Influenza A viruses—i.e., pathogens possibly associated with neurological signs and mortality—78 and 63 samples (represented by cloacal and/or tracheal swabs) from the 92 bird carcasses submitted to the IZSLER tested negative for IAV and NDV, respectively. Only one pooled sample obtained from four unspecified duck carcasses, collected on 9 August 2021, tested positive for IAV. However, following further molecular characterization [36,37,38], this pooled sample tested negative for AIV belonging to the H5 and H7 subtypes. The IAV-positive sample was further characterized at the Italian National Reference Laboratory for Avian Influenza and Newcastle Disease (Legnaro, PD) as an H3N8 antigenic subtype.
- As shown in Table 1, in the 2019–2022 study period, eligible samples (represented by the liver and/or intestine) from the 74 birds tested at the IZLER were examined to confirm the presumptive diagnosis of avian botulism by Multiplex Real-Time PCR (n. 74) and a Mouse Test (n. 22).

**Table 1:** Botulism Confirmatory Resultsthe Multiplex Real-Time PCR and Mouse Test were used to detect BoNT-producing clostridia and BoNT, respectively.

Recovery Data			Samples Submitted to Laboratories	No. Tested/ Submitted	RT-PCR pos./Tested (*Clostridium* Type) *organ*	Mouse Test (pos./Tested)
yy	dd/mm	Locality				
2019	5 September	VM	unspecified ducks	7/7	1/7 * (C) liv. ** and int. ***	n.d.
	5 October	VM	unspecified ducks	4/4	1/4 (CD) *liv.* and *int.*	0/1 ^^ *liv.*
	8 October	VM	pied avocets (*Recurvirostra avosetta*)	9/12	3/9 (C) *liv.* and *int.*	1/1 ^^ *liv.*
		VM	feral pigeon (*Columba livia domestica*)	1/1	0/1 *liv.* and *int.*	n.d.
		VM	Eurasian magpie (*Pica pica*)	1/1	1/1 (C) *liv.* and *int.*	n.d.
	9 October	VM	pied avocets (*R. avosetta*)	2/5	1/2 (C) *liv.* and *int.*	n.d.
2020	17 June	VM	great cormorant (*Phalacrocorax carbo*)	1/1	0/1 *liv.* and *int.*	n.d.
	13 July	PA	mallard (*Anas platyrhynchos*)	1/1	1/1 (C/D) *liv.* and *int.*	0/1 ^^ *liv.*
	19–20 August	VM	unspecified ducks	16/16	1/16 * (C) *int.* ** 0/16 * *liv.* ***	0/16 *^^ *int.*
						n.d.
2021	17–18 August	VM	green-winged teals (*Anas crecca*)	2/2	1/2 (C), 1/2 (C/D) *liv.*	n.d.
					2/2 (C) *int.* ^§^	n.d.
		VM	unspecified ardeid	1/1	0/1 *liv.*	n.d.
					0/1 *int.*	
		VM	mallard (*A. platyrhynchos*)	1/1	1/1 (C/D) *liv.*	1/1 ^^ *liv.*
					1/1 (C/D) *int.*	1/1 ^^ *int.*
		VM	unspecified ducks	9/17	2/9 (C) *liv.*	0/2 ^^ *liv.*
					1/9 (C), 2/9 (C/D) *int.* ^§§^	n.d.
	19 August	VM	unspecified ducks #	4/5	1/4 * *int.* C	n.d.
					0/4 * *liv.*	
	20 August	VM	water samples	10/10	7/10 (C)	n.d.
	25 August	VM	unspecified ducks	2/2	0/2 * *liv.*	n.d.
					1/2 * (C/D) *int.*	n.d.
2022	4 May	PA	mallard (*A. platyrhynchos*)	4/7	1/4 (C), 1/4 (C/D) ^§§§^ *liv.*	n.d.
	3 August	VM	mute swan (*Cygnus olor*)	1/1	0/1 *liv.*	n.d
	9 August	VM	northern shovelers (*S. clypeata*)	3/3	0/3 * *liv.* 0/3 * *int.*	n.d.
		VM	mute swan (*C. olor*)	1/1	1/1 (C/D) *liv.*	n.d.
	10 August	VM	northern shoveler (*S. clypeata*)	1/1	0/1 *liv.*	n.d.
		VM	mallard (*A. platyrhynchos*)	1/1	1/1 (C/D) *liv.* 1/1 (C/D) *int.*	n.d.
		VM	green-winged teal (*A. crecca*)	1/1	0/1 *liv.*	n.d.
	12 August	VM	mute swan (*C. olor*)	1/1	0/1 *liv.*	n.d.

VM, Valle Mandriole (Ravenna Province, northeastern Italy); PA, Punte Alberete (Ravenna Province, northeastern Italy); *, pooled samples (a positive result refers to at least one positive sample); **, liver; ***, intestine; n.d., not done; *^^, sample tested RT-PCR-negative; ^^, sample tested RT-PCR-positive; #, see Section 3.3 for duck species details; ^§^, two green-winged teals tested RT-PCR-positive, as follows: one for the C gene from both the intestine and liver, one for the C and C/D genes from the intestine and liver, respectively. ^§§^, only four of the nine tested ducks were RT-PCR-positive, as follows: two for the C/D gene from the intestine only, one for the C gene from the liver only, one for the C gene from both the intestine and liver; ^§§§^, only two of the four tested mallards were positive, as shown.

- Annual data showed that 7/24, 2/18, 9/19, and 4/13 birds were RT-PCR-positive for BoNT-producing clostridia in 2019, 2020, 2021, and 2022, respectively. When also tested by a Mouse Test, 1/2, 0/17, and 1/3 birds were positive for BoNT in 2019, 2020, and 2021, respectively.
- In addition, 7/10 water samples collected on 20 August, 2021 tested positive for *Clostridium* spp. type C.
- The in-depth bacterial characterization showed the presence of *Clostridium* spp. type C and C/D in the study area.
- Further details on the sampling periods, recovery data, samples examined, and pooled samples are shown in Table 1.

### 3.3. Impact of Avian Botulism on Wild Birds

#### 3.3.1. 2019

- After the first recognition, on 5 September 2019, of 7 wild duck pooled carcasses found to be positive for BoNT-producing clostridia by RT-PCR (Table 1), the avian botulism emergency in VM showed a dramatic escalation in October 2019.
- From 4 October to 11 October, a total of 2158 bird carcasses and 209 birds showing symptoms consistent with avian botulism were collected and managed by suitable, urgent, interventions, as previously described in Section 3.1.
- As shown in Table 2, the number of birds (both dead and ill birds) collected in this period showed a decreasing trend that spanned from 1663 to 19 carcasses and from 180 to 5 symptomatic birds on 4–5 October and 11 October, respectively. Overall, 91.8% of the collected birds (2173/2367) belonged to the Anseriformes order, followed by unspecified wild birds (6.9%, 163/2367), and birds belonging to the orders of Charadriiformes (1.2%, 25/2367), Gruiformes (0.1%, 3/2367), Pelecaniformes (0.04%, 1/2367), Columbiformes (0.04%, 1/2367), and Passeriformes (0.04%, 1/2367). All the Anseriformes birds were accounted for by six dabbling duck species, mainly represented by *Anas crecca* (2031/2173).

**Table 2:** Estimated numbers of wild birds found dead or alive during the large outbreak of avian botulism that occurred in October 2019 in northeastern Italy (Valle Mandriole wetland).

Bird Species	Collected Bird Carcasses (CBC) No.			Collected Symptomatic Birds (CSB) No.			Total No.	
	4–5 October	8–9 October	11 October	4 October	8 October	11 October	CBC	CSB
*Anas crecca*	1452	449	13	97	18	2	1914	117
*Anas platyrhynchos*	48	15	2	11	2	2	65	15
*Spatula clypeata*	20	6	1	12	1	1	27	14
*Mareca strepera*	8	3	1	―	―	―	12	―
*Anas acuta*	2	1	―	2	―	―	3	2
*Mareca penelope*	2	1	―	―	1	―	3	1
*Recurvirostra avosetta*	12	5	―	1	―	―	17	1
*Numenius arquata*	1	―	―	―	―	―	1	―
*Tringa erythropus*	3	―	1	―	1	―	4	1
*Limosa limosa*	―	―	―	―	1	―	―	1
*Rallus aquaticus*	2	―	―	―	―	―	2	―
*Gallinula chloropus*	―	―	1	―	―	―	1	―
*Threskiornis aethiopicus*	1	―	―	―	―	―	1	―
*Columba livia domestica*	―	1	―	―	―	―	1	―
*Pica pica*	―	1	―	―	―	―	1	―
unspecified waterbird	82	24	―	57	―	―	106	57
**Total No.**	1633	506	19	180	24	5	2158	209

―, not reported; *A. crecca*, Green-winged Teal; *A. platyrhynchos*, Mallard; *S. clypeata*, Northern Shoveler; *M. strepera*, Gadwall, *A. acuta*, Northern Pintail; *M. Penelope*, Eurasian Wigeon; *R. avosetta*, Pied Avocet; *N. Arquata*, Eurasian Curlew; *T. erythropus*, Spotted Redshank; *L. limosa*, Black-tailed Godwit; *R. aquaticus*, Water Rail; *G. chloropus*, Moorhen; *T. aethiopicus*, African Sacred Ibis; *C. livia domestica*, feral pigeon; *P. pica*, Eurasian magpie.

- During the carcass collection activities, the advanced decomposition state of a large portion of the collected waterbirds, frequently mixed in the collection tanks with bodies in good condition, underlies the relatively large, estimated number of undetermined bird species.
- Regarding the symptomatic birds, treated as previously described in Section 3.1.1, 57 of the 180 waterbirds showing a variable intoxication degree were soon dead, and no species identification data are available. Based on the 11 October census results, around ten waterbirds (including teals, mallards, gadwalls, and shovelers) showed mild paralysis signs, enabling them to move away by swimming or finally even taking off in flight, thus suggestive of mild or resolving intoxication. Concurrently, less than ten ducks were seen in greater difficulty, and five of them were collected (two teals, two mallards, one shoveler) to be hospitalized at the WTRC aviaries.
- On 29 November 2019, ISPRA gave a positive opinion for the rapid release of recovered birds before the onset of colder weather. The individuals that survived (Table 2) after the first hospitalization at the Ca’ Ponticelle WTRC aviaries (San Vitale pinewood, 44°27′38.52″ N, 12°13′17″ E) were moved to aviaries at the Classe pinewood (south of Ravenna, 44°21′17″ N, 12°17′25″ E) and then released in the nearby protected area of Ortazzo (44°21′00″ N, 12°18′14″ E).
- Further 2019 results on the avian botulism confirmatory tests, sampling days, and bird species collected are shown in Table 1 and Table 2.

#### 3.3.2. 2020

- Of the 17 cases of suspected avian botulism that occurred in VM in 2020, only one pooled sample obtained from 16 duck carcasses tested positive for Type C *Clostridium* spp., and one mallard carcass found on 13 July in the PA area tested Type C/D *Clostridium* spp.-positive (Table 1).

#### 3.3.3. 2021

- When, on 17 August, the first signs of avian botulism were reported in the same VM area affected in 2019, a large-scale operation was then arranged to search for and promptly remove carcasses, as well as to collect sick birds. As shown in Table 3, from 17 August to 25 August, a total of 82 dead birds and 94 birds showing symptoms consistent with avian botulism were collected to remove the potentially dangerous carcasses and treat the sick birds by rehydrating and antibiotic therapy, as previously described in Section 3.1.1. Overall, 97.7% of the collected birds (172/176) belonged to the Anseriformes order, followed by Pelecaniformes (1.7%, 3/176) and birds belonging to the order of Charadriiformes (0.6%, 1/176). Except for a pochard and an unspecified duck, the Anseriformes birds were accounted for by six dabbling duck species, mainly represented by *Anas crecca* (114/172).
- On 25 August, when the last search for botulism-affected birds was carried out, seven symptomatic birds, including six ducks and one little egret, plus two dead teals were collected. No dead or sick birds were found in the following days.
- Further 2021 results on the avian botulism confirmatory tests, sampling days, and bird species collected are shown in Table 1 and Table 3.

#### 3.3.4. 2022

- Two juvenile mallards (estimated age approx. 20–30 days) found on May 5 at the PA wetland (Table 1) were positive for *Clostridium* spp. bacteria capable of producing BoNT toxins: one for type C and the other one for the mosaic variant C/D. However, the presence of emaciation combined with parasitic proventriculitis, compatible with infestation by *Eustrongylides* spp. (further details available upon request), and, most importantly, the absence of environmental conditions necessary to trigger an outbreak of avian botulism accounted for the young mallards’ role of indicators of environmental presence of BoNT-producing clostridia.
- During the mid-summer of 2022, the seven ducks showing symptoms consistent with avian botulism and taken to the local WTRC to be treated as previously described (Section 3.1.1, Section 3.1.3, and Section 3.1.4) included three northern shovelers and one green-winged teal recovered on 9 August, one green-winged teal recovered on August 10, and two green-winged teals recovered on 11 August.
- The nine waterbirds found dead at VM between 3 August and 12 August and submitted to the IZSLER for analyses included three mute swans, four northern shovelers, one mallard, and one green-winged teal. Among these, one mallard and two mute swans tested positive for avian botulism (Table 1).

## 4. Discussion

Avian botulism is a feared neurological disease affecting wild aquatic birds and can occur with different regularity and severity in wetlands of the Americas, Europe, Oceania, Africa, and Asia [6,7]. Although the BoNT intoxication has long been known in North America, this is probably an underestimated phenomenon in Europe, where despite various reports [4,8,14,15,16,46,47,48,49,50,51], avian botulism represents an emerging health problem for which it is difficult assess the extent [1,47].

The ecology of *C. botulinum* is very complex, and the mortality cases, usually very localized, indicate that various concomitant environmental conditions may contribute to the onset of the disease. Indeed, decaying organic material of various origins—such as plants, invertebrate and vertebrate organisms, and, notably, animal carcasses [52]—may constitute a suitable substrate for the vegetative replication of *C. botulinum* strains capable of producing the lethal toxin concomitantly with high temperatures and the absence of oxygen [2,9]. The direct ingestion of *C. botulinum* replication substrates or of invertebrates fed on these substrates—such as carrion fly larvae (so-called maggots) capable of concentrating neurotoxins produced in decomposing carcasses—represents a possible source of intoxication of various waterfowl species [12] showing varying degrees of vulnerability, as described in Spain during avian botulism outbreaks [14]. In this context, the disease creates a unique effect among intoxications because the BoNT produced within the dead birds can lead to the secondary poisoning of other birds. Thus, from an epidemiological point of view, avian botulism could be considered an infectious disease that is able to spread by the carcass–maggot cycle, enabling these invertebrates—not susceptible to the toxin but instead capable of concentrating it [13]—to feed on contaminated carcasses and amplify the intoxication spread when ingested by additional birds, causing carcass accumulation [53,54]. Moreover, the spores produced allow the anaerobic clostridia to survive for years in a quiescent state of life [2,9], and this strategy of environmental resistance accounts for the recurrent nature of avian botulism outbreaks in some areas [55].

All the above ecological factors can represent driving forces for the occurrence and re-occurrence of avian botulism outbreaks in wild waterbirds and need be considered in the management of the disease by the mitigation of ongoing outbreaks and/or the prevention of avian botulism in wetland habitats suitable for clostridia vegetative replication. Thus, during botulism outbreaks, it is very important to act promptly and remove the organic substrates enabling the proliferation of BoNT-producing clostridia, such as bird carcasses [7]. In addition, especially in areas formerly affected by severe outbreaks of avian botulism—so called “botulism-prone areas”, contaminated by clostridia spores and frequently characterized by sporadic but recurrent cases—it is crucial to counteract the predictable onset of outbreaks by the timely implementation of preventive surveillance programs designed to reduce BoNT production and the following exposure of birds, possibly implemented in a time range spanning between 10 and 15 days before and after the expected “botulism season” [7,54].

Consequently, during the occurrence of outbreaks, as well as in botulism endemic areas at risk of possible outbreaks, the flooding or drying of wetlands represent crucial tools for environment management, respectively aimed at preventing (similar to the dispersal of birds from the area) the access of waterbirds to the preformed toxin during an outbreak and at reducing the production of the toxin in botulism-prone areas [54]. Anyway, to achieve the expected effects, both the use of flooding—reducing the availability of the toxic substrate but also, most importantly, counteracting the water anoxia—and the use of drying—making the area unattractive to birds—are subordinate to the skill of moving large volumes of water rapidly [7] and to the awareness that such management options take place in the broader context of the ongoing ecosystem change, including global warming and extreme dry and wet events [56,57].

During the investigation of the large waterbird outbreak occurring in VM in 2019, the clinical signs of the affected birds, the epidemiological background, and the laboratory results allowed us to make a diagnosis of avian botulism. In the context of the habitat degradation occurring in VM since 1996, the driving forces underlying the regression of several wetland habitats of European interest had come into play. In particular, the low water level and the insufficient freshwater supply, aggravated by the weather conditions and modest flow rate of small rivers in the area, made it difficult to counteract the eutrophication of the water and the intrusion of the saline wedge [18,20,58]. These conditions—exacerbated by the introduction of some animal and plant invasive alien species, such as the coypu (*Myocastor coypus*), yellow-bellied slider turtle (*Trachemys scripta*), wels catfish (*Silurus glanis*), black bullhead (*Ameiurus melas*), red swamp crayfish (*Procambarus clarkii*), red-root flatsedge (*Cyperus erythrorhizos*), and water primrose (*Ludwigia peploides*)—accounted for the extensive habitat loss and the rarefaction or disappearance of tens of freshwater animal and plant species in this wetland area [31,59].

To the best of our knowledge, this is the first time that a large outbreak of avian botulism, involving thousands of wild waterbirds, is reported in Italian wetlands by a piece of scientific literature. The total dimensional count of live and dead birds recovered in the October 2019 emergency amounts to 2367 subjects (mostly represented by carcasses), and these numbers probably underestimate the real prevalence of the disease. In fact, a previous study carried out in Canada showed that the numbers of waterfowl found dead in a marsh during a botulism outbreak represented only a subset of birds actually killed by the disease [60]. Except for a pigeon and a magpie, all the samples collected in VM were waterbirds, and among these, dabbling ducks accounted for 91.8% of the total (2173/2367). The high percentage of green-winged teals (2031/2173) allows us to suppose that the low water level at VM provided a suitable foraging niche for this small dabbling duck and the resulting exposure to the toxin available mostly at the sediment surface [61]. After the quick collection of dead and sick birds (4–11 October period) and the flooding of the VM wetland (5–12 October period), the botulism emergency decreased over time in 2019.

The following year, two water inputs were planned at the end of May and in July 2020 in order to provide water levels high enough to prevent the risk of avian botulism in VM during the summer. Only the pooled sample of 16 carcasses from ducks found that year in VM tested positive for clostridia type C by real-time PCR.

- However, despite the efforts made in July 2021 to maintain the water level in VM between 30 and 10 cm a.m.s.l., the reduced hydric availability combined with the increasing temperature and bacterial proliferation in the water triggered a new botulism event starting on 17 August and leading (until 25 August) to the collection of 176 waterbirds (82 carcasses and 94 sick ducks) in the same area affected by the severe outbreak in 2019. On 17 August 2021, to face the botulism spread, a rapid and complete desiccation of the wetland started to promote the removal of fish biomass and deter, also using gas cannons, birds from the contaminated area. The VM desiccation, including a phase of the simultaneous inlet of new freshwater and discharge of decaying water, finished on 10 September, with the beginning of the re-flooding operations lasting a few weeks. Once again, almost all the collected waterbirds belonged to the Anseriformes order, mostly represented by *Anas* crecca (114/172).

In 2022, after the spring collection in the PA nearby area of a few young mallards that tested positive by RT-PCR for Clostridium types C and C/D but were deemed to be carrier birds of clostridia capable of producing BoNT [11], the summer emergency re-occurred in VM (9 carcasses and 7 sick ducks collected), and once again, it was deemed essential to desiccate VM.

From the above botulism cases, it follows that the lack of available freshwater necessary to maintain a constant water level and oxygenation in the VM wetland is a critical limiting factor in the environmental management of the area. Therefore, in the context of any inevitable drying of the area, rapid and total water runoff is recommended, considering that residual pools of water—related to drying and/or rainfall events—could trigger new outbreaks in a botulism-prone area. Moreover, since assigned operators visually recorded the water depth in VM using a water level gauge, it should be considered that the future implementation of smart solutions enabling the real-time monitoring of multiple water quality parameters, including the water level, conductivity, temperature, turbidity, and dissolved oxygen [62], could improve the efficiency of the monitoring process.

Avian botulism outbreaks affecting wild birds throughout Europe are mainly caused by type C/D, shown to be more lethal to birds than type C [63]. Interestingly, in the study area, the molecular characterization of BoNT-producing clostridia showed the co-circulation of both type C and chimeric type C/D. Further molecular studies will be useful in epidemiologically characterizing the clostridia type detected in migratory waterbird populations.

## 5. Conclusions

The ecology of *C. botulinum* is articulated, and the mortality cases, usually very localized, indicate that various concomitant ecological and environmental conditions may contribute to the onset of the disease. Indeed, decaying organic material of various origins—such as vegetables, invertebrate and vertebrate organisms, and, notably, animal carcasses—may constitute a suitable substrate for the vegetative replication of *C. botulinum* strains capable of producing the feared toxin concurrently with high temperatures and the absence of oxygen. The potential risk that human activities have for water quality by contributing to oxygen depletion and the accumulation of organic nutrients, as occurring in the broader area of the Po Delta Biosphere Reserve [64], should also be considered.

Considering the wide range of the above ecological factors, the prevention, management, and control of avian botulism rely crucially on habitat management, the quick and careful collection and removal of carcasses, and the regular monitoring and surveillance of live and dead birds [65,66]. Thus, anthropogenic changes that may trigger the occurrence of avian botulism in wetland habitats [67,68] should be carefully assessed, and habitat management actions including flooding and drying should be adaptively planned and quickly implemented to protect the biodiversity of these vulnerable ecosystems, which are increasingly affected by the effects of ongoing climate change.

## Figures and Tables

**Table 3:** Estimated numbers of waterbirds found dead or alive during the outbreak of avian botulism that occurred in August 2021 in northeastern Italy (Valle Mandriole wetland).

Bird Species	No. of Collected Bird Carcasses (CBC)							No. of Collected Symptomatic Birds (CSB)							Total No.	
	17 August	18 August	19 August	20 August	21 August	22 August	25 August	17 August	18 August	19 August	20 August	21 August	22 August	25 August	CBC	CSB
*Anas platyrhynchos*	14	2	―	―	―	―	―	9	―	2	2	1	―	1	16	15
*Anas crecca*	35	18	2	―	―	―	2	29	16	4	2	2	1	3	57	57
*Spatula querquedula*	2	―	―	―	―	―	―	2	―	―	―	―	―	1	2	3
*Spatula clypeata*	―	1	―	―	―	―	―	5	―	―	―	―	―	―	1	5
*Mareca strepera*	―	―	―	―	―	―	―	8	―	1	―	1	―	―	―	10
*Aythya ferina*	―	―	―	―	―	―	―	―	―	―	―	―	―	1	―	1
*Anas acuta*	―	―	―	―	―	―	―	1	―	―	―	―	―	―	―	1
Unspecified duck	1	―	3	―	―	―	―	―	―	―	―	―	―	―	4	―
*Egretta garzetta*	―	―	―	―	―	―	―	1	―	―	―	―	―	1	―	2
*Plegadis falcinellus*	1	―	―	―	―	―	―	―	―	―	―	―	―	―	1	―
*Himantopus himantopus*	―	1	―	―	―	―	―	―	―	―	―	―	―	―	1	―
**Total No.**	53	22	5	0	0	0	2	55	16	7	4	4	1	7	82	94

―, not reported; *A. platyrhynchos*, Mallard; *A. crecca*, Green-winged Teal; *S. querquedula*, Garganey; *S. clypeata*, Northern Shoveler; *M. strepera*, Gadwall; *A. ferina*, Commun Pochard; *A. acuta*, Northern Pintail; *E. garzetta*, Little Egret; *P. falcinellus*, Glossy Ibis; *H. himantopus*, Black-winged Stilt.

## Data Availability

The original contributions presented in the study are included in the article; further inquiries can be directed to the corresponding author.

## Appendix A

To better analyze the epidemiological situation and promptly implement suitable and urgent interventions to counteract the avian botulism emergency, the following key elements were previously acquired by the Technical Table:

1. the VM water supply networks were for multiple uses, starting with drinking and industrial use;
2. in 2019, characterized by a very rainy May, water was supplied by Ravenna Servizi Industriali society (RSI) and released in VM from 17 July to 9 August; after the latter date (as notified by RSI), maintenance works began on the so-called “canaletta ANIC”, the pipeline built in the 1950s by the Azienda Nazionale Idrogenazione Combustibili society to take water from the Reno River to the industrial area; therefore, it was no longer possible to supply water;
3. never in the past had there been a need to turn on the water flow at the beginning of October, and the prolonged and anomalously high temperatures in September probably favored the onset of the problem;
4. the control measures based on draining these areas in summer and cleaning them of vegetation were at the moment difficult to implement (as the area did not dry and the mud could attract waders); thus, the inflow of water from the Reno River to the VM was planned for the week of 5–12 October 2019;
5. removing as many carcasses as possible quickly and treating symptomatic subjects (e.g., with rehydration and antibiotic therapy) were needed actions;
6. precautional measures to be implemented included the use of gloves when handling both dead and symptomatic animals, as well as the respect of the most common hygiene standards.

Technical Appendix A. Key elements acquired by the Technical Table of experts, healthcare and local administration personnel, and stakeholders during the 2019 botulism emergency in the Valle Mandriole protected area (Ravenna Province, Emilia Romagna Region, northeastern Italy).
